# A retrospective, matched case-control study of recombinant LH versus hMG supplementation on FSH during controlled ovarian hyperstimulation in the GnRH-antagonist protocol

**DOI:** 10.3389/fendo.2022.931756

**Published:** 2022-08-15

**Authors:** Ming-Jer Chen, Yu-Chiao Yi, Hwa-Fen Guu, Ya-Fang Chen, Hsiao-Fan Kung, Jui-Chun Chang, Shih-Ting Chuan, Li-Yu Chen

**Affiliations:** ^1^ Department of Obstetrics and Gynecology and Women’s Health, Taichung Veterans General Hospital, Taichung, Taiwan; ^2^ School of Medicine, National Yang Ming Chiao Tung University, Taipei, Taiwan

**Keywords:** controlled ovarian hyperstimulation, recombinant FSH, recombinant LH, human menopausal gonadotrophin (hMG), pregnancy

## Abstract

**Background:**

The role of luteinizing hormone (LH) in controlled ovarian hyperstimulation (COH) requires more evidence for its efficacy. Several studies compared recombinant human LH (r-hLH) or human menopausal gonadotropin (hMG) in combination with recombinant human follicle-stimulating hormone (r-hFSH) but lack the results with GnRH-antagonist protocol and in Asians.

**Methods:**

This is a retrospective, single-center study inspecting women receiving GnRH antagonist protocol and r-hFSH+hMG or r-hFSH+r-hLH regimen for over five days for COH in the *in vitro* fertilization (IVF)/intracytoplasmic sperm injection (ICSI) cycle in Taiwan from 2013 to 2018. The outcomes of IVF/ICSI cycles were analyzed after propensity score matching between the two groups. A subgroup analysis was conducted in cycles in which women underwent their first embryo transfer (ET), including fresh ET and frozen ET (FET).

**Results:**

With a total of 503 cycles, the results revealed that the r-hFSH+r-hLH group performed better in terms of numbers of oocytes retrieved (r-hFSH+hMG vs. r-hFSH+r-hLH, 11.7 vs. 13.7, p=0.014), mature oocytes (8.7 vs. 10.9, p=0.001), and fertilized oocytes (8.3 vs. 9.8, p=0.022), while other outcomes were comparable. The analysis of first ET cycles also showed similar trends. Although the implantation rate (39% vs. 43%, p=0.37), pregnancy rate (52% vs. 53%, p=0.90), and live birth rate (39% vs. 45%, p=0.19) were not significantly different, the miscarriage rate was higher in the r-hFSH+hMG group than the r-hFSH+r-hLH group (26% vs. 15%, p<0.05) in first ET cycles. The cumulative live birth rate was significantly higher in the r-hFSH+r-hLH group (53% vs. 64%, p=0.02). No significant difference in rates of ovarian hyperstimulation syndrome (OHSS) was observed.

**Conclusion:**

The results support the hypothesis that the treatment of r-hLH+r-hFSH improves COH clinical outcomes in the IVF/ICSI cycle.

## Introduction

Controlled ovarian hyperstimulation (COH) in the *in vitro* fertilization (IVF)/intracytoplasmic sperm injection (ICSI) cycle is dependent on the regulation of gonadotropin-releasing hormone (GnRH) and gonadotropin treatment. Among the gonadotropins, follicle-stimulating hormone (FSH) is necessary to stimulate follicle growth in the ovaries, while the role of luteinizing hormone (LH) remains debatable. A meta-analysis suggests no significant difference between FSH+LH and FSH alone with GnRH-antagonist protocol in COH (1), but the supplement of LH appears to be beneficial in certain subgroups of women in more recent studies (2–4). Notably, LH supplement was shown to lead to a higher cumulative live birth rate (cLBR) than FSH alone in poor ovarian responders in a large retrospective study containing more than 9,000 cycles (5). In clinical practice, human menopausal gonadotropin (hMG) with both FSH and LH bioactivity is often applied for COH. Even though highly purified hMG suffers from impurity and potential contaminants due to being extracted from human urine (6), it is reported that the effects of hMG and recombinant human follicle-stimulating hormone (r-hFSH) are comparable in COH (7, 8).

Several studies compared the use of hMG versus r-hFSH plus recombinant human luteinizing hormone (r-hLH) but with discrepant results. Overall, there was no significant difference in pregnancy rates observed in the total population (9–11). Two studies revealed that the groups receiving r-hFSH+r-hLH had a higher pregnancy rate than hMG in patients with hypogonadotropic hypogonadism or with poor ovarian reserve (12, 13). Another study demonstrated that the pregnancy rate was significantly higher in the group of r-hFSH+r-hLH when the oocyte yield was high (9). As for the safety concerns, two reports indicated a higher risk of ovarian hyperstimulation syndrome (OHSS) when patients are treated with r-hFSH+r-hLH than with hMG (10, 11).

Other research has been conducted to compare the effects of r-hLH and hMG in the presence of FSH. A retrospective study found that the group of r-hFSH+r-hLH had more oocytes retrieved, more embryos, and a higher pregnancy rate than that of r-hFSH+hMG (14). Similar outcomes were demonstrated by a randomized controlled trial: a higher number of mature oocytes and higher pregnancy rates were observed in the group supplementing r-hLH than its hMG counterpart (15). One particular three-group study compared the efficacy of r-hFSH+r-hLH, r-hFSH+hMG, and hMG alone (16). Among the three groups, the r-hFSH+r-hLH one performed best in clinical pregnancy rate and implantation rate. Overall, previous studies suggested that r-hLH resulted in better outcomes than hMG when applied in addition to FSH.

The aforementioned studies mostly focused on Western populations, and none of them have provided evidence of the treatment outcomes of r-hFSH+hMG in Asians. Besides, only a few of them applied GnRH-antagonist protocol, which was reported to have lower risks of OHSS without significant difference in clinical pregnancy rate and live birth rate comparing to GnRH-agonist protocol for COH (17, 18). A meta-analysis of subgroup analyses stratified by GnRH-agonist or antagonist protocols found evidence that the outcomes of assisted reproductive technology (ART) may differ between protocol types (19). In consequence, we conducted a real-world study to evaluate the efficacy of r-hFSH+r-hLH versus r-hFSH+hMG in COH with GnRH-antagonist protocol for the IVF/ICSI cycle in Taiwanese patients managed in our center.

## Methods

### Study design

**Figure 1:**
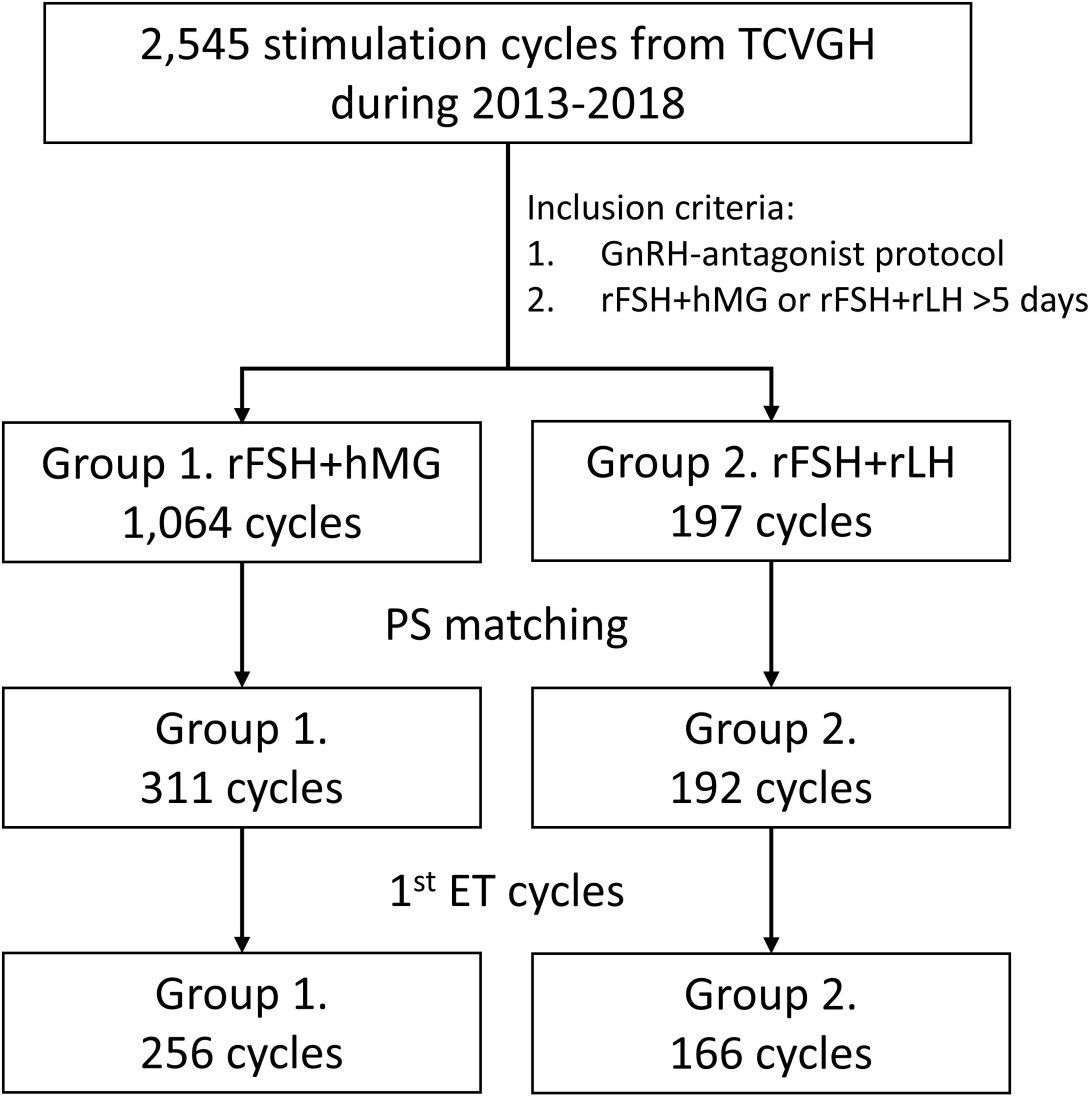
Flowchart of the study design.

The data were collected from women who received COH regimens between 2013 and 2018 in Taichung Veterans General Hospital (TCVGH, Taiwan). The selection criteria included those who received GnRH-antagonist protocol and were treated with the r-hFSH+r-hLH or r-hFSH+hMG regimen for at least 5 days before further procedures. The flowchart of the study design is presented in **Figure 1**. A total of 1,064 cycles in Group 1 (r-hFSH+hMG) and 197 cycles in Group 2 (r-hFSH+r-hLH) meeting the criteria were included. Propensity score matching was applied to select matched subjects with balanced age, anti-mullerian hormone (AMH) level, and oocyte retrieval date across the two groups with a ratio of 2:1. After matching, 311 cycles in Group 1 and 192 cycles in Group 2 were analyzed. Since the best embryos were transferred at the first embryo transfer (ET), the cycles in which patients received their first fresh ET or frozen ET (FET) procedure were further subjected to subgroup analysis, including 256 cycles in Group 1 and 166 cycles in Group 2. The outcomes were estradiol (E2) levels on the human chorionic gonadotropin (hCG)-administered day, endometrial thickness on the hCG-administered day, number of oocytes retrieved, number of mature oocytes, number of fertilized oocytes, number of grade 1 to 2 embryos on cycle day 3, number of embryos transferred, number of successful implantations, implantation rate, pregnancy rate, live birth rate, miscarriage rate, the rate of OHSS, and cLBR. The study was approved by the institutional review board of TCVGH (Permission number: CE21401B).

### Ovarian stimulation and oocyte retrieval

Baseline levels of serum FSH and LH were tested on cycle day 2 or 3. In Group 1, the COH was executed through r-hFSH (Gonal-F, 75–300 IU per day) and hMG (Menopur, 75–300 IU FSH and 75–300 IU LH per day), while in Group 2, patients received r-hFSH and r-hLH (Pergoveris, 150–300 IU FSH and 75–150 IU LH per day) either with or without the supplement of r-hFSH (Gonal-F, 75–300 IU per day). GnRH antagonist (Cetrotide, 0.25 mg/day) was administered for pituitary gland downregulation with a flexible protocol starting when the dominant follicle developed to 12–14 mm in diameter until the day of hCG trigger. When at least 3 dominant follicles were larger than 17 mm in average diameter, recombinant hCG (Ovidrel, 250 μg) and/or GnRH agonist (Decapeptyl, 0.2 mg) was delivered to trigger ultimate oocyte maturation. The dual trigger with Ovidrel and Decapeptyl has been routinely performed since 2014 in our center, except for the patients with high risks of OHSS, who were triggered with GnRH agonist only and whose fresh cycle were canceled. Oocyte retrieval was conducted 35–37 hours after the trigger.

### Fertilization, embryo transfer, and pregnancy assessment

The oocytes were inseminated using IVF or ICSI. The embryos were graded according to the morphological classification by Veeck’s criteria (20). One to four fresh or frozen embryos were transferred per cycle. Luteal support was given from the day after oocyte retrieval with a combination of oral Duphaston 10 mg BID and transvaginal 8% Crinone gel 90 mg QD until the day of hCG check-up and continued to 8 gestational weeks (GWs) in fresh ET cycles. In FET cycles, the endometrial preparations were programmed either by hormone replacement cycles or modified natural cycles, depending on the individual conditions of each patient. Details of the endometrium priming and luteal phase support have been reported earlier (21). Pregnancy was confirmed with the positive β-hCG serum level tested 14 days after oocyte retrieval. Implantation was defined as the visible gestational sacs in 6 GWs. Live birth was defined as the delivery of a live baby after at least 24 GWs. Cumulative live birth was defined as the occurrence of live birth from one oocyte retrieval cycle until all the available embryos from that cycle were used. The second live birth from the same oocyte retrieval cycle would not be counted again.

### Statistics

Greedy nearest neighbor matching was applied to calculate the propensity score matching in baseline parameters in a ratio of 2:1 for Group 1 and 2. Categorical items were described using percentages and frequencies, whereas continuous characteristics were assessed using descriptive statistics (number of subjects, mean, standard deviation). The parameters were compared using two-sample t-test for continuous variables, and the comparison between categorical variables was conducted with chi-square test. Normality tests were not performed for continuous data due to the assumption of a sufficient sample size (n=503). Significance was defined as P-value <0.05. All analyses were processed with SAS^®^ version 9.4 or later (SAS Institute Inc, Cary, NC, USA).

## Results

The patients in Group 1 or Group 2 were treated with r-hFSH+hMG or r-hFSH+r-hLH during COH, respectively. The baseline characteristics of the total analyzed cycles are shown in **Table 1**. The mean ages of the patients were 36.05 and 35.48 years, with the body mass index (BMI) around 22.5 and the average anti-mullerian hormone (AMH) level 3.5 and 3.8 ng/ml. The age, BMI, and AMH levels in both groups were comparable after propensity score matching (p>0.05). The validity of the matching process was supported by the similarity in the infertility history (p=0.801), causes of infertility (p=0.343), and basal FSH level (Group 1 vs. Group 2, 7.1 vs. 7.4 mIU/mL, p=0.485). The basal LH level was slightly higher in Group 2 (3.9 vs. 4.6 mIU/mL, p=0.034), but the values were within the reference range in both groups. The trigger types were significantly different between the two groups (p=0.001). The analysis showed that the total dose of FSH and LH were significantly lower (p<0.001) in Group 2 than in Group 1, and that the induction duration was shorter in Group 2 (10.22 vs. 9.8 days, p=0.003) as well. In short, with similar baseline characteristics except for basal LH level and trigger type, a lower total dose of gonadotropins was used in the r-hFSH+r-hLH group. No significant difference was seen in the ratio of FSH to LH dose between the two groups.

**Table 1:** Baseline characteristics of total stimulation cycles after matching.

	GROUP 1. r-hFSH + hMG	GROUP 2. r-hFSH + r-hLH	P-value
**Stimulation cycle**	**n=311**	**n=192**	
Age (year)	36.05 ± 3.56	35.48 ± 3.90	0.099
BMI (kg/m^2^)	22.48 ± 3.51	22.30 ± 3.15	0.562
AMH (ng/mL)	3.53 ± 3.49	3.77 ± 3.51	0.448
Infertility history			0.801
Primary	186 (59.81%)	117 (60.94%)	
Secondary	125 (40.19%)	75 (39.06%)	
Causes of infertility			0.343
Uterine factor	89 (28.62%)	40 (20.83%)	
Ovarian factor	81 (26.05%)	47 (24.48%)	
Tubal factor	29 (9.32%)	24 (12.50%)	
Male factor	26 (8.36%)	22 (11.46%)	
Mix factor	23 (7.40%)	16 (8.33%)	
Others	63 (20.26%)	43 (22.40%)	
Basal FSH (mIU/mL)	7.13 ± 3.16	7.35 ± 3.67	0.485
Basal LH (mIU/mL)	3.88 ± 2.06	4.58 ± 4.19	0.034*
Total FSH dose (IU)	3362.45 ± 1072.79	2602.15 ± 930.26	<0.001*
Total LH dose (IU)	1152.01 ± 608.85	947.66 ± 510.60	<0.001*
FSH/LH dose ratio	4.12 ± 4.18	4.05 ± 3.85	0.843
Induce days	10.22 ± 1.83	9.80 ± 1.31	0.003*
Oral agents (used) ^@^	5 (1.61%)	5 (2.60%)	0.437
Growth hormone (used)	13 (4.18%)	6 (3.13%)	0.547
Trigger type ^#^			0.001*
Dual trigger ^a^	272 (87.46%)	153 (79.69%)	
GnRH-agonist ^b^	17 (5.47%)	27 (14.06%)	
r-hCG ^c^	15 (4.82%)	12 (6.25%)	
Others	7 (2.25%)	0 (0.00%)	
Procedure ^$^			0.411
IVF ^d^	203 (65.27%)	117 (60.94%)	
ICSI ^e^	88 (28.30%)	56 (29.17%)	
IVF+ICSI	14 (4.50%)	11 (5.73%)	
TESE-ICSI ^f^	6 (1.93%)	8 (4.17%)	

*Statistical significance. Values are presented in mean ± standard deviation or number (percentage). ^@^ Oral agents: Letrozole or Clomiphene ^#^ a) Dual trigger = Ovidrel 250 μg + Decapeptyl 0.2 mg; b) GnRH-agonist = Decapeptyl 0.2 mg; c) r-hCG = Ovidrel 500 μg ^$^ d) IVF: in vitro fertilization; e) ICSI: Intracytoplasmic Sperm Injection; f) TESE: Testicular Sperm Extraction

The outcomes of the IVF/ICSI cycles are shown in **Table 2**. The E2 level on the hCG-administered day was significantly higher in Group 2 (2468 vs. 2909 pg/ml, p=0.022). No significant difference was observed in endometrial thickness and the number of grade 1 to 2 embryos on day 3. The numbers of oocytes retrieved (11.7 vs. 13.7, p=0.014), mature oocytes (8.7 vs. 10.9, p=0.001), and fertilized oocytes (8.3 vs. 9.8, p=0.022) were higher in Group 2 than in Group 1. The rates of moderate or severe OHSS were similar between the two groups (p=0.969). To summarize, r-hFSH+r-hLH performed better than or equal to r-hFSH+hMG in the stimulatory outcomes examined.

**Table 2:** Outcomes of total stimulation cycles after matching.

	GROUP 1. r-hFSH +hMG	GROUP 2. r-hFSH + r-hLH	P-value
**Stimulation cycle**	**n=311**	**n=192**	
E2 level on the trigger day (pg/mL)	2467.92 ± 1730.82	2908.87 ± 2265.30	0.022*
Endometrial thickness (mm)	11.18 ± 2.76	11.09 ± 2.58	0.722
No. of oocytes retrieved	11.65 ± 8.78	13.69 ± 9.43	0.014*
No. of mature oocytes	8.71 ± 6.97	10.92 ± 7.88	0.001*
No. of fertilized oocytes	8.25 ± 7.05	9.81 ± 7.86	0.022*
No. of Grade 1-2 embryos on Day 3	2.74 ± 3.27	3.06 ± 3.37	0.293
Moderate or severe OHSS	5 (1.61%)	3 (1.56%)	0.969

*Statistical significance. Values are presented in mean ± standard deviation or number (percentage).

Similar results were obtained when investigating the cycles in which patients underwent their first ET. Most baseline characteristics did not significantly differ (p>0.05), while the total dose of gonadotropins and induce days were less in Group 2 (**Table S1**). The basal serum level of LH was higher in Group 2 (3.9 vs. 4.7 IU/L, p=0.032), and the trigger types between the two groups showed a significant difference (p=0.007). The outcomes of the first ET cycles are presented in **Table 3**. Among the outcomes before the ET, the E2 level on the trigger day (2633 vs. 3079 pg/ml, p=0.036), the numbers of oocytes retrieved, mature oocytes, and fertilized oocytes were superior in Group 2. The endometrial thickness and the number of grade 1 to 2 embryos on day 3 were not significantly different (p=0.665 and p=0.302, respectively). As for the outcomes after the ET, the implantation rate (39% vs. 43%, p=0.37), the pregnancy rate (52.3% vs. 53.0%, p=0.893), and the live birth rate (38.7% vs. 45.2%, p=0.185) were higher in Group 2 than in Group 1 even though not significantly different, and the miscarriage rate was significantly lower in Group 2 (26.1% vs. 14.8%, p=0.045). The risks of OHSS were comparable (p=0.848). Based on the IVF/ICSI and ET results, the r-hFSH+r-hLH group achieved better performance not only on some of the parameters before the ET but also on the miscarriage rate in the first ET cycles.

**Table 3:** Outcomes of 1^st^ ET cycles.

	GROUP 1. r-hFSH + hMG	GROUP 2. r-hFSH+ r-hLH	P-value
**Stimulation cycle**	**n=256**	**n=166**	
E2 level on the trigger day (pg/ml)	2633.41 ± 1771.69	3079.41 ± 2313.80	0.036*
Endometrial thickness (mm)	11.29 ± 2.80	11.18 ± 2.57	0.665
No. of oocytes retrieved	12.54 ± 8.81	14.64 ± 9.53	0.021*
No. of mature oocytes	9.38 ± 6.86	11.87 ± 7.89	<0.001*
No. of fertilized oocytes	9.21 ± 7.14	10.82 ± 7.88	0.031*
No. of Grade 1-2 embryos on Day 3	3.12 ± 3.36	3.47 ± 3.44	0.302
No. of embryos transferred	2.11 ± 0.75	1.95 ± 0.80	0.030*
No. of successful implantation	0.71 ± 0.77	0.70 ± 0.76	0.896
Implantation rate	39 ± 44%	43 ± 46%	0.370
Pregnancy rate	134 (52.34%)	88 (53.01%)	0.893
Live birth rate	99 (38.67%)	75 (45.18%)	0.185
Miscarriage rate	35 (26.12%)	13 (14.77%)	0.045*
Moderate or severe OHSS	4 (1.56%)	3 (1.81%)	0.848

*Statistical significance. Values are presented in mean ± standard deviation or number (percentage).

**Figure 2:**
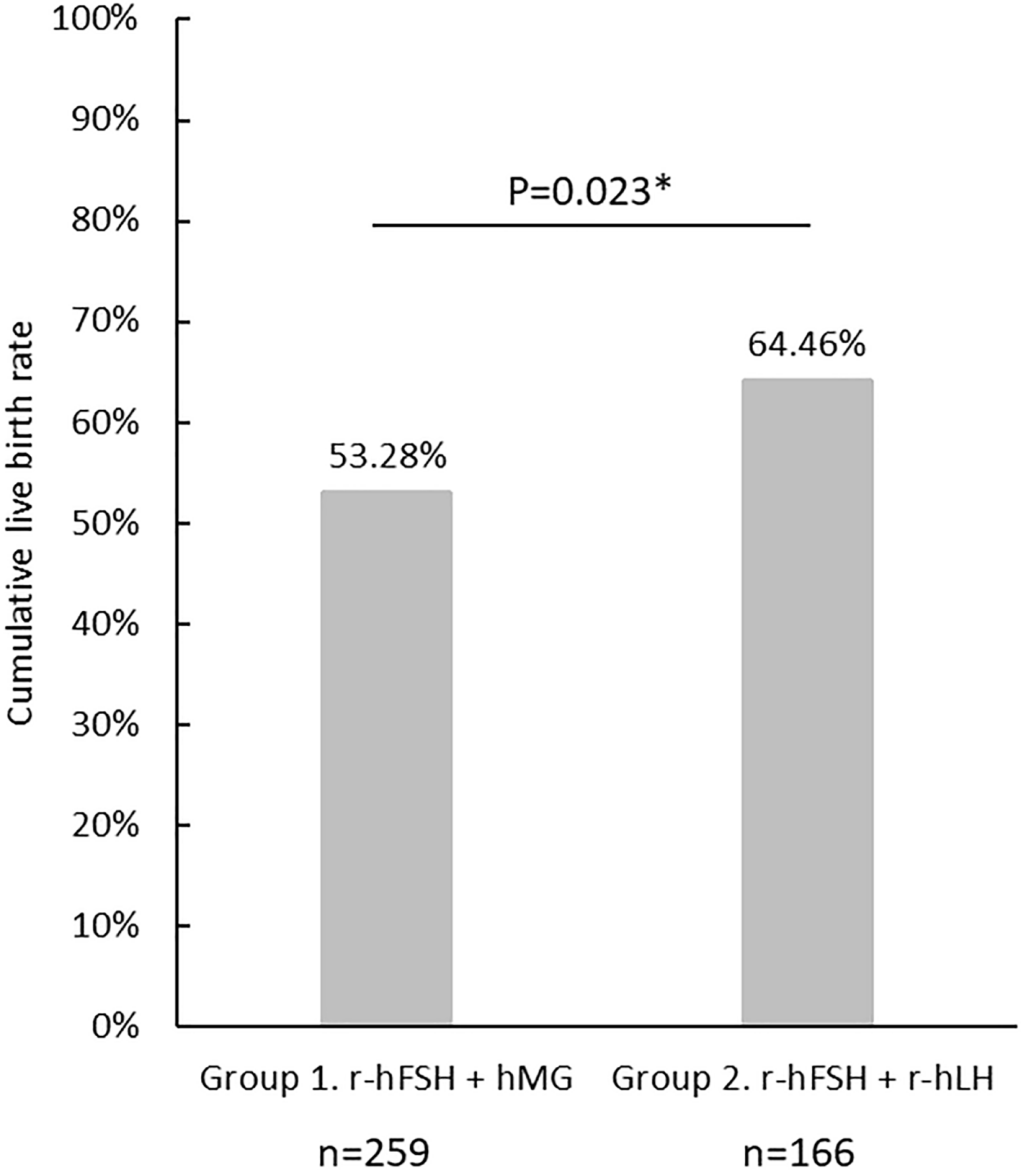
The cumulative live birth rate in Group 1(r-hFSH+hMG) and Group 2 (r-hFSH+r-hLH). *represents statistical significance (p<0.05).

The cLBR of the cycles included in the analysis is shown in **Figure 2**. There were 138 cycles out of 259 achieved live births in Group 1 (cLBR = 53.3%), and 107 out of 166 in Group 2 (cLBR = 64.5%). The cLBR was significantly higher in the r-hFSH+r-hLH group than in the r-hFSH+hMG group (p=0.023).

To avoid potential bias caused by the fresh or frozen ET procedures, we divided the subgroups of the first ET cycles into fresh ET and FET groups. The baseline characteristics and outcomes are presented in **Tables S2**, **S3** and **4**, **5**. In the fresh ET subgroup, there were 123 cycles in Group 1 and 71 cycles in Group 2. Most of the baseline characteristics were not significantly different except the total FSH dose (Group 1 vs. Group 2, 3226 vs. 2532, p<0.001) (**Table S2**). The numbers of oocytes retrieved, mature oocytes, fertilized oocytes, and grade 1–2 embryos on day 3 were higher in Group 2 (**Table 4**). The observation agreed to the results of total cycles or the first ET cycles although lack statistical significance. In the FET subgroup, 133 cycles in Group 1 and 95 cycles in Group 2 were included. The total FSH dose, total LH dose, and induce days were significantly lower in Group 2 (**Table S3**). The number of mature oocytes was higher in Group 2 (15.9 vs. 18.0, p=0.007), and the numbers of oocytes retrieved, fertilized oocytes and grade 1–2 embryos on day 3 showed a similar trend with the fresh ET subgroup (**Table 5**). Overall, the patterns of the fresh ET or FET subgroup analyses were consistent with those of total cycles or the first ET cycles.

**Table 4:** Outcomes of 1^st^ fresh ET cycles.

	GROUP 1. r-hFSH +hMG	GROUP 2. r-hFSH + r-hLH	P-value
**Stimulation cycle**	**n=123**	**n=71**	
E2 level on the trigger day (pg/mL)	1844.60 ± 1192.00	1922.61 ± 1170.59	0.660
Endometrial thickness (mm)	11.15 ± 2.92	11.18 ± 2.73	0.945
No. of oocytes retrieved	8.94 ± 5.91	10.13 ± 6.30	0.191
No. of mature oocytes	6.80 ± 4.50	8.11 ± 5.51	0.075
No. of fertilized oocytes	6.54 ± 4.56	7.34 ± 4.92	0.258
No. of Grade 1-2 embryos on Day 3	2.31 ± 2.18	2.55 ± 2.58	0.491
No. of embryos transferred	2.20 ± 0.83	2.06 ± 0.84	0.265
No. of successful implantation	0.47 ± 0.63	0.54 ± 0.71	0.520
Implantation rate	27 ± 39%	31 ± 44%	0.470
Pregnancy rate	49 (39.84%)	30 (42.25%)	0.741
Live birth rate	34 (27.64%)	25 (35.21%)	0.270
Miscarriage rate	15 (30.61%)	5 (16.67%)	0.167
Moderate or severe OHSS	1 (0.81%)	2 (2.82%)	0.276

Values are presented in mean ± standard deviation or number (percentage).

**Table 5:** Outcomes of 1^st^ FET cycles.

	GROUP 1. r-hFSH + hMG	GROUP 2. r-hFSH+ r-hLH	P-value
**Stimulation cycle**	**n=133**	**n=95**	
E2 level on the trigger day (pg/ml)	3356.49 ± 1906.57	3953.16 ± 2572.19	0.058
Endometrial thickness (mm)	11.43 ± 2.68	11.18 ± 2.46	0.466
No. of oocytes retrieved	15.87 ± 9.71	18.02 ± 10.14	0.107
No. of mature oocytes	11.76 ± 7.76	14.68 ± 8.24	0.007*
No. of fertilized oocytes	11.68 ± 8.16	13.42 ± 8.65	0.122
No. of Grade 1-2 embryos on Day 3	3.87 ± 4.03	4.16 ± 3.83	0.590
No. of embryos transferred	2.04 ± 0.67	1.86 ± 0.77	0.069
No. of successful implantation	0.94 ± 0.82	0.83 ± 0.77	0.315
Implantation rate	50 ± 45%	52 ± 46%	0.800
Pregnancy rate	85 (63.91%)	58 (61.05%)	0.660
Live birth rate	65 (48.87%)	50 (52.63%)	0.576
Miscarriage rate	23 (23.53%)	8 (13.79%)	0.150
Moderate or severe OHSS^†^	3 (2.26%)	1 (1.05%)	0.495

*Statistical significance. Values are presented in mean ± standard deviation or number (percentage). ^†^The OHSS occurred in previous fresh cycles on the same patient.

## Discussion

The present study aimed to investigate the clinical outcomes of r-hFSH+hMG versus r-hFSH+r-hLH during COH in women receiving antagonist protocol. Both r-hFSH+hMG and r-hFSH+r-hLH groups achieved similar results in endometrial thickness, embryo development, and implantation rate in the IVF/ICSI cycle. Nevertheless, the r-hFSH+r-hLH regimen performed significantly better in terms of E2 level, oocytes retrieval, and numbers of mature or fertilized oocytes. The r-hFSH+r-hLH group attained better clinical outcomes with a lower total dose of gonadotropin than the r-hFSH+hMG group while no significant difference in the rate of OHSS was present. The cLBR was significantly higher in the r-hFSH+r-hLH group, which agreed to the results of pregnancy rates in previous similar studies (14, 15). A meta-analysis regarding multiple combinations of gonadotropins on ART also indicated the highest pregnancy rate in the group administering r-hFSH with r-hLH than hMG or FSH alone (19). The results of the present study implied that LH activity from purified or recombinant sources could lead to discrepant outcomes in COH. The efficacy of r-hLH may be more consistent than hMG when used to stimulate LH receptors, as Dahan et al. suggested (14). Besides, it was reported that LH and hCG evoked different cellular and molecular responses, albeit acting on the same receptor (i.e., luteinizing hormone-chorionic gonadotropin receptor [LHCGR]) (22). Since the LH activity in hMG was mainly from its hCG component, which has a longer half-life in the human body than LH and shares only ~85% identity with LH (23), it is not surprising that the two regimens led to significantly different results.

Aside from the outcomes generally investigated by other studies, we particularly analyzed the cycles of the first ET (both fresh ET and FET) regarding that the best embryos were chosen at the first ET attempt. Moreover, from the patients’ points of view, getting pregnant at the first ET shortened the duration of treatment. In terms of the parameters before the embryo transfer procedure, the patterns of the outcomes in the first ET cycles were comparable to those in total cycles, with better clinical outcomes including the numbers of retrieved, mature, and fertilized oocytes in the r-hFSH+r-hLH group. Fewer embryos were transferred with a significantly lower miscarriage rate in the group of r-hFSH+r-hLH. There was also a consistent trend of higher implantation rate, pregnancy rate, and live birth rate in the r-hFSH+r-hLH group, although not significantly different. Giving the live birth rate differed by 6.5% (Group 1 vs. Group 2, 38.7% vs. 45.2%), the small sample size may have played a role in the lack of statistical significance. In conclusion, r-hFSH+r-hLH showed more positive outcomes than r-hFSH+hMG in the analysis of either total cycles or the first ET cycles.

The lower total FSH and LH dose in the r-hFSH+r-hLH group than the r-hFSH+hMG group is consistent with the data in previous investigations with similar study groups (14, 16). The patients benefit from not only less exposure to medications but also from fewer burdens of injection treatment. This phenomenon was not observed in the study conducted by Gómez-Palomares et al., in which only women over 38 years old were recruited (15). However, in the study of Bühler and Fischer, the significantly lower dose of the r-hFSH+r-hLH group was seen in both subgroups of over and under 35 years old (16). As a result, more information is needed to identify whether the discrepancy among the literature in gonadotropin dose was due to age or other factors.

The research on the efficacy of hMG versus FSH in COH has been abundant (19). Nonetheless, during retrospective data collection, we found that hMG or r-hFSH was seldomly used alone in the hospital except for women with distinct baseline characteristics. The group of r-hFSH alone were relatively younger (mean age = 33.72) and had a higher AMH level (mean = 6.91 ng/ml), whereas the group of hMG alone tended to be older (mean age = 39.27) with a lower AMH level (mean = 1.11 ng/ml). Consequently, this study focused on the r-hFSH+hMG and r-hFSH+r-hLH groups for analysis. The two combinations were frequently seen in real-world practice according to the clinical data and were hence meaningful to help provide insights in determining a suitable regimen for COH. To our knowledge, this study is the first to compare hMG and r-hLH when supplemented to r-hFSH during COH with GnRH-antagonist protocol on Eastern Asians. Previous studies comparing r-hFSH+hMG and r-hFSH+r-hLH were mainly conducted on Western populations, and most of them applied GnRH-agonist protocol for pituitary gland downregulation (14–16). There have been few studies in Asian countries, and the design of their research groups differs from the present study (24, 25).

While most baseline parameters between the study groups were similar, the basal LH level and the trigger types were statistically different in this study. A previous study suggested no significant correlation between the basal LH level and IVF outcomes (26). Higher basal LH levels were reported to be associated with better ART outcomes in another study, and the LH levels were 3.4 ± 0.7 mIU/mL and 7.2 ± 2.8 mIU/mL in the literature (27). In our study, the values were much closer. The basal LH level of Group 1 was 3.88 ± 2.06 mIU/mL in total cycles and 3.93 ± 2.03 mIU/mL in the first ET cycles, and the number was 4.58 ± 4.19 mIU/mL and 4.72 ± 4.42 mIU/mL in Group 2, respectively. All these mean values of the basal LH levels were within the reference range. The difference in LH levels between the two groups was therefore considered acceptable. Concerning the trigger types, a recent meta-analysis that included both the GnRH agonist and the dual trigger versus the hCG found no significant difference in pregnancy rate (28). The number of oocytes retrieved was reported to be significantly higher with the dual trigger than hCG alone. Given that the vast majority applied the dual trigger and only a small proportion applied hCG alone in our study, we suggest that the trigger type would not be a critical factor affecting the results when comparing hMG and r-hLH.

The study was limited by its retrospective nature. Additionally, this study utilized propensity score matching to reduce the bias caused by demographic factors or baseline characteristics, which may result in some limitations to the analysis. First, the basal LH level and the trigger types were statistically different as mentioned above. Secondly, the matching method produced an average population for analysis, while outliers in age, AMH level, basal follicle count, or other factors were excluded. As many other studies examined patients with poor ovarian reserve, women of higher age, or poor responders, the current study could not achieve the same objectives. Finally, this was a single-center study, which limited the sample size and scope of the analysis. The matching process eliminated a large proportion of cycles in the r-hFSH+hMG group, further reducing the sample size from one thousand to around three hundred. The algorithm planned to create sample sizes for Groups 1 and 2 in a 2:1 ratio, but the eventual ratio was closer to 3:2, reflecting the restraint on the sample size. In brief, the matching case-control rather than randomization was the main limitation as a retrospective study.

Collectively, the clinical outcomes of COH with r-hFSH+r-hLH were either equal or superior to those with r-hFSH+hMG both in the total cycles and first ET cycles with no significant difference in the risks of OHSS. The results suggest that when administered GnRH-antagonist protocol in Taiwanese women, the r-hFSH+r-hLH regimen could lead to higher cLBR. The finding of the present study adds to the increasing evidence of better clinical outcomes of r-hFSH+r-hLH than r-hFSH+hMG in COH. Future research is needed to corroborate this finding and to contribute to the understanding of COH for ART treatment.

## Data availability statement

The raw data supporting the conclusions of this article are available from the corresponding author on reasonable request, without undue reservation.

## Ethics statement

The studies involving human participants were reviewed and approved by The Institutional Review Board of Taichung Veterans General Hospital No.1650, Sec.4, Taiwan Boulevard, Taichung City, Taiwan. Written informed consent for participation was not required for this study in accordance with the national legislation and the institutional requirements.

## Author contributions

M-JC and Y-CY contributed to conception and design of the study. H-FG, Y-FC, H-FK, J-CC, S-TC, and L-YC were responsible for the acquisition of data and organization of the database. Y-FC analysed the data. M-JC and Y-CY drafted the article. All authors contributed to manuscript revision, read, and approved the submitted version.

## Funding

Medical writing assistance was funded by Merck Ltd., an affiliate of Merck Healthcare KGaA, Darmstadt, Germany.

## Conflict of interest

The authors declare that this study received funding from Merck Ltd., an affiliate of Merck Healthcare KGaA, Darmstadt, Germany. The funder had the following involvement with the study: writing assistance.

## Publisher’s note

All claims expressed in this article are solely those of the authors and do not necessarily represent those of their affiliated organizations, or those of the publisher, the editors and the reviewers. Any product that may be evaluated in this article, or claim that may be made by its manufacturer, is not guaranteed or endorsed by the publisher.
