# Corrigendum: A retrospective, matched case-control study of recombinant LH versus hMG supplementation on FSH during controlled ovarian hyperstimulation in the GnRH-antagonist protocol

**DOI:** 10.3389/fendo.2022.1099647

**Published:** 2022-12-07

**Authors:** Ming-Jer Chen, Yu-Chiao Yi, Hwa-Fen Guu, Ya-Fang Chen, Hsiao-Fan Kung, Jui-Chun Chang, Shih-Ting Chuan, Li-Yu Chen

**Affiliations:** ^1^Department of Obstetrics and Gynecology and Women’s Health, Taichung Veterans General Hospital, Taichung, Taiwan; ^2^School of Medicine, National Yang Ming Chiao Tung University, Taipei, Taiwan

**Keywords:** controlled ovarian hyperstimulation, recombinant FSH, recombinant LH, human menopausal gonadotrophin (hMG), pregnancy

In the published article, there was an error. Instead of “cumulative pregnancy rate”, it should be “cumulative live birth rate”.

A correction has been made to the Synopsis. This sentence previously stated:

“The cumulative pregnancy rate was significantly higher in the r-hFSH+r-hLH group (53% vs. 64%, p=0.02).”

The corrected sentence appears below:

“The cumulative live birth rate was significantly higher in the r-hFSH+r-hLH group (53% vs. 64%, p=0.02).”

In the published article, there was an error. Instead of “patients”, it should be “cycles”.

A correction has been made to Section 3 Results. The sentences previously stated:

“The cLBR of the patients included in the analysis is shown in **Figure 2**. There were 138 patients out of 259 achieved live births in Group 1 (cLBR = 53.3%), and 107 out of 166 in Group 2 (cLBR =64.5%).”

**Figure 2:**
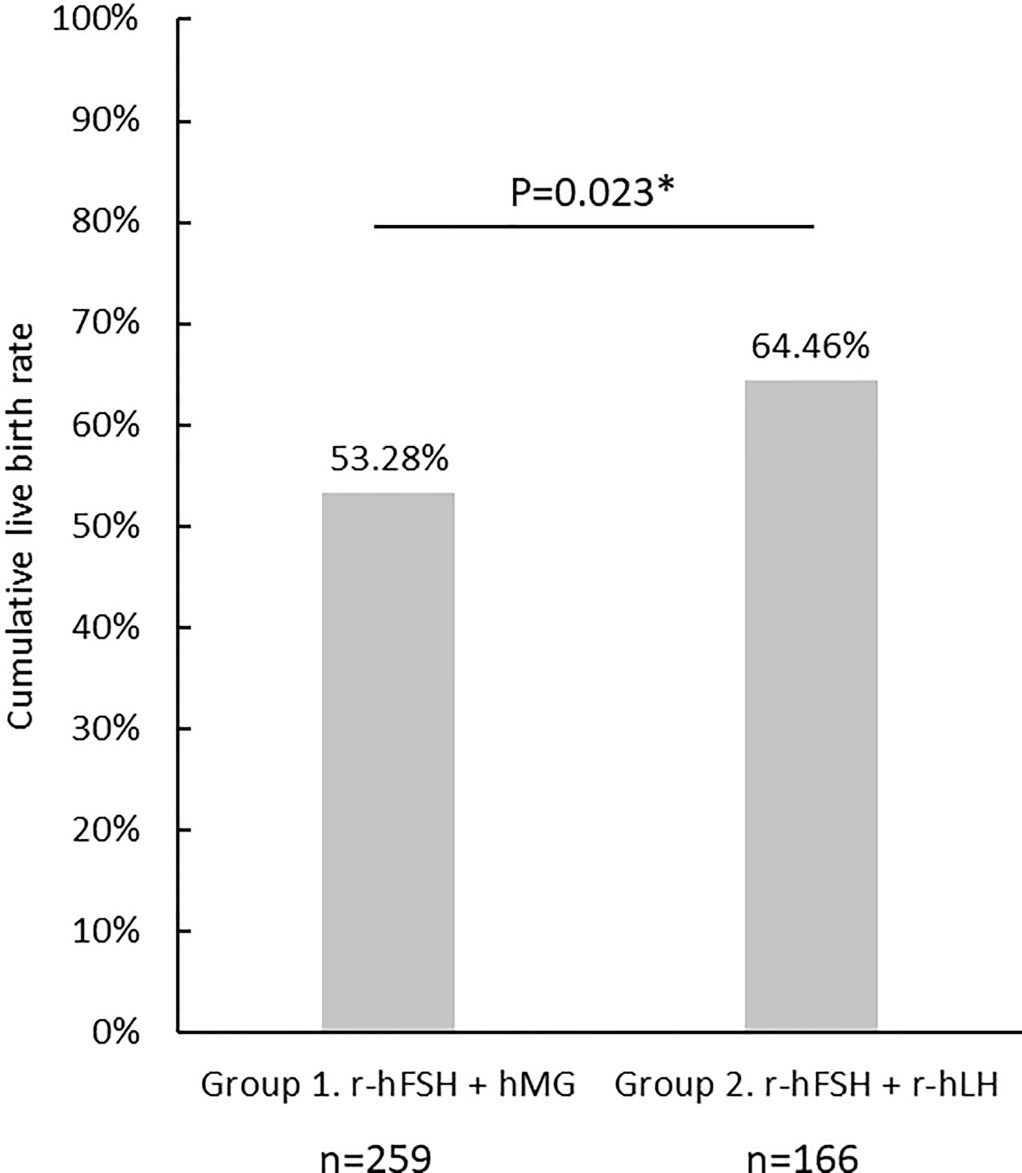
The cumulative live birth rate in Group 1 (rFSH+hMG) and Group 2 (rFSH+rLH). "*"represents statistical significance (p<0.05).

The corrected sentence appears below:

“The cLBR of the cycles included in the analysis is shown in **Figure 2**. There were 138 cycles out of 259 achieved live births in Group 1 (cLBR = 53.3%), and 107 out of 166 in Group 2 (cLBR =64.5%).”

In the published article, there was an error in **Figure 2** as published. The y-axis title was marked “Cumulative pregnancy rate”. The corrected y-axis title of **Figure 2** should be “Cumulative live birth rate”.

The corrected figure and its caption appear below:

The authors apologize for the errors and state that these do not change the scientific conclusions of the article in any way. The original article has been updated.

## Publisher’s note

 All claims expressed in this article are solely those of the authors and do not necessarily represent those of their affiliated organizations, or those of the publisher, the editors and the reviewers. Any product that may be evaluated in this article, or claim that may be made by its manufacturer, is not guaranteed or endorsed by the publisher.

