# Supplementary material for: A retrospective, matched case-control study of recombinant LH versus hMG supplementation on FSH during controlled ovarian hyperstimulation in the GnRH-antagonist protocol
Source: Front Endocrinol (Lausanne). 2022 Aug 15;13:931756. doi: 10.3389/fendo.2022.931756 (PMC9420867; doi:10.3389/fendo.2022.931756)
Supplement: Supplementary file 1 [file DataSheet_1.docx]

**Supplementary Material**

Supplementary Tables:

**Table S1. Baseline characteristics of 1^st^ ET cycles.**

|  | **GROUP 1.**  **r-hFSH + hMG** | **GROUP 2.**  **r-hFSH+ r-hLH** | **P-value** |
| --- | --- | --- | --- |
| **Stimulation cycle** | **n=256** | **n=166** |  |
| Age (year) | 35.73 ± 3.38 | 35.32 ± 3.73 | 0.242 |
| BMI (kg/m^2^) | 22.38 ± 3.52 | 22.28 ± 3.23 | 0.772 |
| AMH (ng/mL) | 3.78 ± 3.62 | 4.08 ± 3.63 | 0.408 |
| Infertility history |  |  | 0.916 |
| Primary | 154 (60.16%) | 99 (59.64%) |  |
| Secondary | 102 (39.84%) | 67 (40.36%) |  |
| Causes of infertility |  |  | 0.298 |
| Uterine factor | 68 (26.56%) | 30 (18.07%) |  |
| Ovarian factor | 69 (26.95%) | 40 (24.10%) |  |
| Tubal factor | 29 (11.33%) | 24 (14.46%) |  |
| Male factor | 25 (9.77%) | 20 (12.05%) |  |
| Mix factor | 16 (6.25%) | 14 (8.43%) |  |
| Others | 49 (19.14%) | 38 (22.89%) |  |
| Basal FSH (mIU/mL) | 7.14 ± 3.09 | 7.19 ± 3.62 | 0.884 |
| Basal LH (mIU/mL) | 3.93 ± 2.03 | 4.72 ± 4.42 | 0.032* |
| Total FSH dose (IU) | 3303.76 ± 1078.26 | 2516.34 ± 883.29 | <0.001* |
| Total LH dose (IU) | 1115.63 ± 624.68 | 909.94 ± 508.31 | <0.001* |
| FSH/LH dose ratio | 4.36 ± 4.54 | 4.18 ± 4.05 | 0.672 |
| Induce days | 10.27 ± 1.75 | 9.77 ± 1.24 | <0.001* |
| Oral agents (used) ^@^ | 4 (1.56%) | 3 (1.81%) | 0.848 |
| Growth hormone (used) | 9 (3.52%) | 5 (3.01%) | 0.778 |
| Trigger type ^#^ |  |  | 0.007* |
| Dual trigger ^a^ | 220 (85.94%) | 130 (78.31%) |  |
| GnRH-agonist ^b^ | 17 (6.64%) | 25 (15.06%) |  |
| r-hCG ^c^ | 13 (5.08%) | 11 (6.63%) |  |
| Others | 6 (2.34%) | 0 (0.00%) |  |
| Procedure ^$^ |  |  | 0.535 |
| IVF ^d^ | 171 (66.80%) | 106 (63.86%) |  |
| ICSI ^e^ | 66 (25.78%) | 42 (25.30%) |  |
| IVF+ICSI | 14 (5.47%) | 11 (6.63%) |  |
| TESE-ICSI ^f^ | 5 (1.95%) | 7 (4.22%) |  |
| ET type |  |  | 0.288 |
| Fresh cycle | 123 (48.0%) | 71 (42.8%) |  |
| FET | 133 (52.0%) | 95 (57.2%) |  |

*Statistical significance. Values are presented in mean ± standard deviation or number (percentage).

^@^ Oral agents: Letrozole or Clomiphene

^#^ a) Dual trigger = Ovidrel 250 μg + Decapeptyl 0.2 mg; b) GnRH-agonist = Decapeptyl 0.2 mg; c) r-hCG = Ovidrel 500 μg

^$^ d) IVF: *in vitro* fertilization; e) ICSI: Intracytoplasmic Sperm Injection; f) TESE: Testicular Sperm Extraction

**Table S2. Baseline characteristics of 1^st^ fresh ET cycles**

|  | **GROUP 1.**  **r-hFSH + hMG** | **GROUP 2.**  **r-hFSH + r-hLH** | **P-value** |
| --- | --- | --- | --- |
| **Stimulation cycle** | **n=123** | **n=71** |  |
| Age (year) | 36.23 ± 3.14 | 35.55 ± 3.86 | 0.210 |
| BMI (kg/m^2^) | 21.94 ± 3.27 | 22.06 ± 3.17 | 0.818 |
| AMH (ng/mL) | 3.02 ± 3.10 | 2.96 ± 2.98 | 0.889 |
| Infertility history |  |  | 0.801 |
| Primary | 70 (56.91%) | 42 (59.15%) |  |
| Secondary | 53 (43.09%) | 29 (40.85%) |  |
| Causes of infertility |  |  | 0.343 |
| Uterine factor | 29 (23.58%) | 12 (16.90%) |  |
| Ovarian factor | 43 (34.96%) | 23 (32.39%) |  |
| Tubal factor | 12 (9.76%) | 10 (14.08%) |  |
| Male factor | 11 (8.94%) | 10 (14.08%) |  |
| Mix factor | 12 (9.76%) | 7 (9.86%) |  |
| Others | 16 (13.01%) | 9 (12.68%) |  |
| Basal FSH (mIU/mL) | 7.91 ± 3.30 | 7.74 ± 4.74 | 0.789 |
| Basal LH (mIU/mL) | 3.70 ± 1.76 | 4.11 ± 2.32 | 0.198 |
| Total FSH dose (IU) | 3226.43 ± 1021.25 | 2532.57 ± 857.92 | <0.001* |
| Total LH dose (IU) | 1113.41 ± 552.06 | 965.49 ± 493.75 | 0.063 |
| FSH/LH ratio | 3.97 ± 3.60 | 3.81 ± 3.81 | 0.775 |
| Induce days | 9.80 ± 1.57 | 9.46 ± 1.25 | 0.099 |
| Oral agents (used) ^@^ | 3 (2.44%) | 0 (0.00%) | 0.185 |
| Growth hormone (used) | 5 (4.07%) | 2. (2.82%) | 0.653 |
| Trigger type ^#^ |  |  | 0.291 |
| Dual trigger ^a^ | 109 (88.62%) | 65 (91.55%) |  |
| GnRH-agonist ^b^ | 1 (0.81%) | 0 (0.00%) |  |
| r-hCG ^c^ | 8 (6.50%) | 6 (8.45%) |  |
| Others | 5 (4.07%) | 0 (0.00%) |  |
| Procedure ^$^ |  |  | 0.249 |
| IVF ^d^ | 87 (70.73%) | 50 (70.42%) |  |
| ICSI ^e^ | 30 (24.39%) | 14 (19.72%) |  |
| IVF+ICSI | 4 (3.25%) | 2 (2.82%) |  |
| TESE-ICSI ^f^ | 2 (1.63%) | 5 (7.04%) |  |

*Statistical significance. Values are presented in mean ± standard deviation or number (percentage).

^@^ Oral agents: Letrozole or Clomiphene

^#^ a) Dual trigger = Ovidrel 250 μg + Decapeptyl 0.2 mg; b) GnRH-agonist = Decapeptyl 0.2 mg; c) r-hCG = Ovidrel 500 μg

^$^ d) IVF: *in vitro* fertilization; e) ICSI: Intracytoplasmic Sperm Injection; f) TESE: Testicular Sperm Extraction

**Table S3. Baseline characteristics of 1^st^ FET cycles.**

|  | **GROUP 1.**  **r-hFSH + hMG** | **GROUP 2.**  **r-hFSH+ r-hLH** | **P-value** |
| --- | --- | --- | --- |
| **Stimulation cycle** | **n=133** | **n=95** |  |
| Age (year) | 35.27 ± 3.54 | 35.15 ± 3.64 | 0.798 |
| BMI (kg/m^2^) | 22.78 ± 3.71 | 22.45 ± 3.29 | 0.485 |
| AMH (ng/mL) | 4.49 ± 3.92 | 4.92 ± 3.86 | 0.405 |
| Infertility history |  |  | 0.628 |
| Primary | 84 (63.16%) | 57 (60.00%) |  |
| Secondary | 49 (36.84%) | 38 (40.00%) |  |
| Causes of infertility |  |  | 0.361 |
| Uterine factor | 39 (29.32%) | 18 (18.95%) |  |
| Ovarian factor | 26 (19.55%) | 17 (17.89%) |  |
| Tubal factor | 17 (12.78%) | 14 (14.74%) |  |
| Male factor | 14 (10.53%) | 10 (10.53%) |  |
| Mix factor | 4 (3.01%) | 7 (7.37%) |  |
| Others | 33 (24.81%) | 29 (30.53%) |  |
| Basal FSH (mIU/mL) | 6.42 ± 2.69 | 6.78 ± 2.42 | 0.300 |
| Basal LH (mIU/mL) | 4.15 ± 2.24 | 5.18 ± 5.46 | 0.086 |
| Total FSH dose (IU) | 3375.28 ± 1127.54 | 2504.21 ± 906.12 | <0.001* |
| Total LH dose (IU) | 1117.67 ± 687.13 | 868.42 ± 517.61 | 0.002* |
| FSH/LH ratio | 4.73 ± 5.26 | 4.45 ± 4.23 | 0.663 |
| Induce days | 10.70 ± 1.80 | 9.99 ± 1.18 | <0.001* |
| Oral agents (used) ^@^ | 1 (0.75%) | 3 (3.16%) | 0.172 |
| Growth hormone (used) | 4 (3.01%) | 3 (3.16%) | 0.948 |
| Trigger type ^#^ |  |  | 0.030* |
| Dual trigger ^a^ | 111 (83.46%) | 65 (68.42%) |  |
| GnRH-agonist ^b^ | 16 (12.03%) | 25 (26.32%) |  |
| r-hCG ^c^ | 5 (3.76%) | 5 (5.26%) |  |
| Others | 1 (0.75%) | 0 (0.00%) |  |
| Procedure ^$^ |  |  | 0.911 |
| IVF ^d^ | 84 (63.16%) | 56 (58.95%) |  |
| ICSI ^e^ | 36 (27.07%) | 28 (29.47%) |  |
| IVF+ICSI | 10 (7.52%) | 9 (9.47%) |  |
| TESE-ICSI ^f^ | 3 (2.26%) | 2 (2.11%) |  |

*Statistical significance. Values are presented in mean ± standard deviation or number (percentage).

^@^ Oral agents: Letrozole or Clomiphene

^#^ a) Dual trigger = Ovidrel 250 μg + Decapeptyl 0.2 mg; b) GnRH-agonist = Decapeptyl 0.2 mg; c) r-hCG = Ovidrel 500 μg

^$^ d) IVF: *in vitro* fertilization; e) ICSI: Intracytoplasmic Sperm Injection; f) TESE: Testicular Sperm Extraction
